# Incremental cost of state funds for new enrollment in Section 1332 waivers

**DOI:** 10.1093/haschl/qxaf050

**Published:** 2025-03-12

**Authors:** David M Anderson, Daniel Ludwinski

**Affiliations:** Department of Health Services, Policy and Management, University of South Carolina, Columbia, SC 29201, United States; Department of Economics, Oxford College, Emory University, Oxford, GA 30054, United States

**Keywords:** Affordable Care Act, state policy, reinsurance, Section 1332 waiver

## Introduction

The Affordable Care Act (ACA) allows states to modify their state individual health insurance market with Section 1332 waivers. Section 1332 reinsurance lowers unsubsidized premiums in the ACA individual health insurance markets as state funds pay for some high-cost claims.^1^ These lower premiums decrease the cost of premium tax credits for the federal government, which then passes these savings to the state government.^1^ By December 1, 2024, 16 states received Section 1332 waivers from the federal government to enact state-funded reinsurance programs, and all prioritized increasing enrollment in health insurance in their initial applications.

Lowering the premium for health insurance has 2 effects on consumer behavior. First, it reduces costs for existing buyers who do not receive subsidies. This is significant because premium costs for individuals who do not receive federal financial assistance have consistently increased since 2014.^2^ This group benefits from a decrease in their out-of-pocket expenses, which can improve their overall financial stability and access to healthcare services. Second, a lower premium decreases the minimum cost of coverage for individuals who previously found health insurance premiums to be unaffordable. These individuals, who previously would not have purchased insurance due to high costs, may now decide to purchase ACA health insurance. This analysis is focused on this group of newly insured individuals and compares the extra state costs, defined as only the state expenditures without regard to federal pass-through funding, to the expected increase in enrollment.

## Methods

We examined the approved waiver applications filed to the Centers for Medicare and Medicaid Services for 15 states, and 21 waivers, including re-applications (Appendix A2) (To access the Appendix, Click on the Details Tab of the Article Online.).^3^ We excluded the second 2022 Colorado waiver extension application as it implemented a public option, and we excluded Minnesota as their waiver application did not have a state wide average reduction in premium available. We extracted the state's estimate for first-year state costs and incremental enrollment gains. We calculated the state's cost per new enrollee by dividing the state expenditures on the program and the projected net new enrollment with waiver compared with projected enrollment without the waiver. All data is publicly available administrative data.

## Results

**Figure 1.**
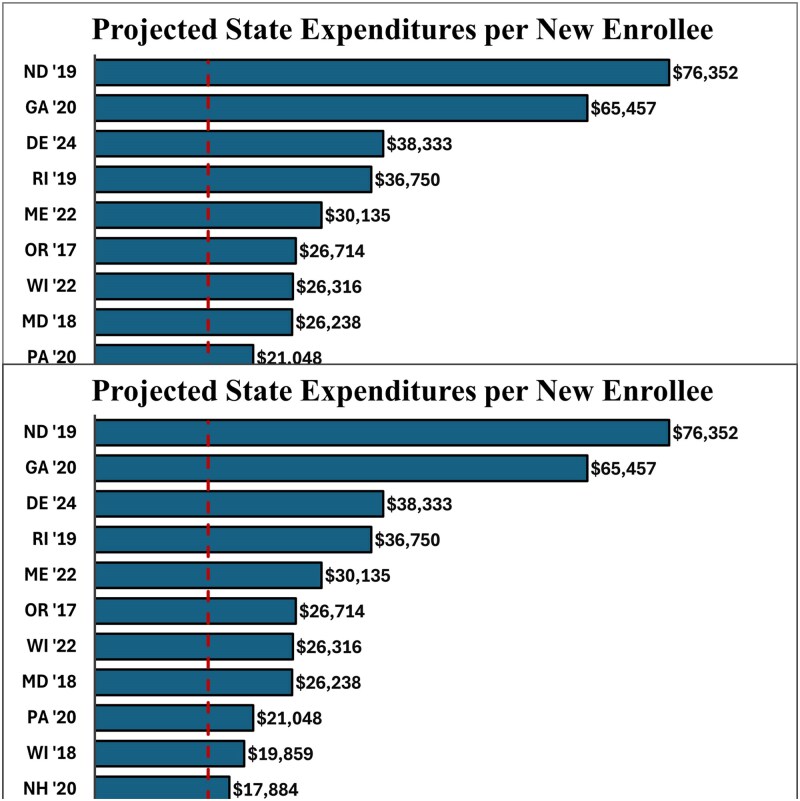
Projected state expenditures per new enrollee in the first year of the waiver. Source: Authors' analysis of final approved state Section 1332 waiver applications.^3^ The authors identified state expenditures without any federal pass-through revenue as the denominator and the projected number of net new enrollees with the waiver relative to the without the waiver counterfactual as the numerator. All dollars are nominal dollars unadjusted for inflation.

The cost per new enrollee has substantial variance across states and years (Figure 1). The enrollment weighted mean state cost per new enrollee was $15 065. The minimum state cost of an extra enrollee was $5091 in Colorado and the maximum state cost was $76 352 in North Dakota. Initial applications for Section 1332 waivers had a weighted average state cost of a new enrollee of $15 844 while waiver renewal applications had a weighted average state cost of a new enrollee of $12 399. The predicted increase in new enrollees varied from 0.4% to 13% with a weighted average of 2.5% and the predicted drop in premiums varied from 4.2% to 40% with a weighted average of 13.7% (Appendix Table A1) (To access the Appendix, Click on the Details Tab of the Article Online).

## Discussion

Section 1332 reinsurance programs are widely implemented by a diverse set of sixteen states. States submit actuarial projections for enrollment changes and state costs to the federal government. All states project enrollment gains among only the individuals who were unlikely to receive federal premium subsidies. However, there are substantial differences in the expected state-funded costs of insuring one additional individual between and within states. These differences were observed for waiver applications submitted both before and after the passage of enhanced and expanded subsidies in the American Rescue Plan Act.^4^ Relative to other programs that are intended to reduce administrative burden of enrollment, such as reminder letters to targeted populations, reinsurance is an expensive way to increase enrollment.^5^

Other work has shown reinsurance in the ACA to be effective but inefficient at reducing variance in costs.^6^ State-funded reinsurance has been shown to lower overall premiums while increasing the minimum cost of coverage for individuals who receive premium subsidies.^1,7^ Recent evidence of all state reinsurance programs find that net premium reductions are roughly equivalent to the reinsurance funds; no positive virtuous cycle emerges.^8^ Our analysis is limited in only examining the actuarial projections of net enrollment gains and state costs; we do not observe post-implementation results, assess the accuracy of the projections, or create any original predictions. Our analysis shows that most of the benefits of reinsurance accrue to individuals who would have already purchased health insurance on the ACA marketplaces rather than encouraging new purchases of health insurance. Therefore, section 1332 reinsurance may be better conceptualized as an indirect state subsidy and transfer payment from the government to higher-income but unsubsidized enrollees.^2^

## Supplementary Material

qxaf050_Supplementary_Data

## References

[1] Oyeka O, Wehby GL. Effects of state reinsurance programs on health insurance exchange premiums and insurer participation. Health Serv Res. 2023;58(5):1077–1088. 10.1111/1475-6773.14205 37488998 PMC10480091

[2] Anderson D, Abraham JM, Drake C. Rural-urban differences in individual-market health plan affordability after subsidy payment cuts. Health Aff (Millwood). 2019;38(12):2032–2040. 10.1377/hlthaff.2019.00917 31794305

[3] Section 1332: State Innovation Waivers | CMS . Center for medicare and medicaid services. Accessed March 10, 2025. https://www.cms.gov/CCIIO/Programs-and-Initiatives/State-Innovation-Waivers/Section_1332_State_Innovation_Waivers-

[4] Meuse D, Levitis J. The American Rescue Plan’s Premium Tax Credit Expansion—State policy considerations. Brookings. April 19, 2021. Accessed March 7, 2025. https://www.brookings.edu/blog/usc-brookings-schaeffer-on-health-policy/2021/04/19/what-does-the-american-rescue-plans-premium-tax-credit-expansion-and-the-uncertainty-around-it-mean-for-state-health-policy/

[5] Goldin J, Lurie IZ, McCubbin J. Health insurance and mortality: experimental evidence from taxpayer outreach. Q J Econ. 2021;136(1):1–49. 10.1093/qje/qjaa029

[6] Polyakova M, Bhatia V, Bundorf MK. Analysis of publicly funded reinsurance—government spending and insurer risk exposure. JAMA Health Forum. 2021;2(8):e211992. 10.1001/jamahealthforum.2021.1992 35977191 PMC8796983

[7] Anderson DM, Golberstein E, Drake C. Georgia's reinsurance waiver associated with decreased premium affordability and enrollment. Health Aff (Millwood). 2024;43(3):398–407. 10.1377/hlthaff.2023.00971 38437604

[8] Hill SC, Jacobs PD. Implications of state reinsurance programs for marketplaces. Am J Health Econ. 10.1086/733523

