# Supplementary material for: Incremental cost of state funds for new enrollment in Section 1332 waivers
Source: Health Aff Sch. 2025 Mar 12;3(4):qxaf050. doi: 10.1093/haschl/qxaf050 (PMC11970019; doi:10.1093/haschl/qxaf050)
Supplement: qxaf050_Supplementary_Data [file qxaf050_supplementary_data.zip › qxaf050_Supplementary_Data.docx]

**Appendix Table A1**

**Table A1: Approved Section 1332 Reinsurance Waiver Characteristics**

| State | Year Filed | Application Type | Actuarial Firm | Reinsurance Type | Total State Cost | # of new enrollees | State Funds per new Enrollee |
| --- | --- | --- | --- | --- | --- | --- | --- |
| Alaska | 2016 | Initial | Oliver Wyman | Designated High Cost Risk Pool | $14.5 M | 1,641 | $8,834 |
| Alaska | 2022 | Renewal | Oliver Wyman | Designated High Cost Risk Pool | $13.5 M | 1,600 | $8,438 |
| Colorado | 2019 | Initial | Lewis and Ellis | Cost Attachment and Ceiling | $87.0 M | 6,378 | $13,641 |
| Colorado | 2021 | Renewal | Lewis and Ellis | Cost Attachment and Ceiling | $34.6 M | 6,806 | $5,091 |
| Delaware | 2019 | Initial | Oliver Wyman | Cost Attachment and Ceiling | $6.9 M | 500 | $13,800 |
| Delaware | 2024 | Renewal | Oliver Wyman | Cost Attachment and Ceiling | $23.0 M | 600 | $38,333 |
| Georgia | 2020 | Initial | Deloitte and Touche | Cost Attachment and Ceiling | $101.0 M | 1,543 | $65,457 |
| Maine | 2022 | Initial | Gorman | Designated High Cost Risk Pool | $26.7 M | 886 | $30,135 |
| Maryland | 2018 | Initial | Wakely | Cost Attachment and Ceiling | $262.3 M | 9,996 | $26,238 |
| Minnesota | 2017 | Initial | Minnesota Department of Insurance | Cost Attachment and Ceiling | $132.7 M | 20,000 | $6,635 |
| New Hampshire | 2020 | Initial | Oliver Wyman | Cost Attachment and Ceiling | $13.4 M | 748 | $17,884 |
| New Jersey | 2018 | Initial | Oliver Wyman | Cost Attachment and Ceiling | $105.8 M | 9,000 | $11,756 |
| New Jersey | 2023 | Renewal | Oliver Wyman | Cost Attachment and Ceiling | $148.5 M | 9,800 | $15,153 |
| North Dakota | 2019 | Initial | Novarest | Cost Attachment and Ceiling | $21.2 M | 278 | $76,352 |
| Oregon | 2017 | Initial | Wakely | Cost Attachment and Ceiling | $90.0 M | 3,369 | $26,714 |
| Oregon | 2022 | Renewal | Novarest | Cost Attachment and Ceiling | $58.6 M | 5,895 | $9,935 |
| Pennsylvania | 2020 | Initial | Oliver Wyman | Cost Attachment and Ceiling | $44.2 M | 2,100 | $21,048 |
| Rhode Island | 2019 | Initial | Wakely | Cost Attachment and Ceiling | $14.7 M | 400 | $36,750 |
| Rhode Island | 2024 | Renewal | Wakely | Cost Attachment and Ceiling | $6.3 M | 400 | $15,675 |
| Wisconsin | 2018 | Initial | Wakely | Cost Attachment and Ceiling | $33.9 M | 1,706 | $19,859 |
| Wisconsin | 2022 | Renewal | Wakely | Cost Attachment and Ceiling | $50.0 M | 1,900 | $26,316 |

Source: Authors’ analysis of final approved state Section 1332 waiver applications.^3^ State funds per new enrollee is calculated by the having total state funds spent (excluding any federal pass-through revenue) in the first year of the waiver divided by the projected increase in total enrollment in the first year in the waiver application.

**Appendix Table A2**

**Table A2: URLs of archived versions of the state waivers**

| State | Year Filed | Application Type | URL |
| --- | --- | --- | --- |
| Alaska | 2016 | Initial | https://web.archive.org/web/20250306145340/https://www.commerce.alaska.gov/web/Portals/11/Pub/Headlines/Alaska%201332%20State%20Innovation%20Waiver%20June%2015%202017.pdf |
| Alaska | 2022 | Renewal | https://web.archive.org/web/20250306145357/https://www.commerce.alaska.gov/web/Portals/11/Pub/INS_1332.WaiverExtension_3.17.22.pdf |
| Colorado | 2019 | Initial | https://web.archive.org/web/20250306145301/https://drive.google.com/file/d/1_QTfHnQvamJWeupH7AScekJe3A_jNo5H/view |
| Colorado | 2021 | Renewal | https://web.archive.org/web/20250306145512/https://drive.google.com/file/d/1niJDFKG6ZtW132_nrMP0LbRu6YNiVSC5/view |
| Delaware | 2019 | Initial | https://web.archive.org/web/20250301170143/https://www.cms.gov/cciio/programs-and-initiatives/state-innovation-waivers/downloads/delaware-1332-waiver-application-july-10-2019.pdf |
| Delaware | 2024 | Renewal | https://web.archive.org/web/20250306144808/https://www.cms.gov/files/document/delaware-section-1332-waiver-extension-application-202405-full.pdf |
| Georgia | 2020 | Initial | https://web.archive.org/web/20250306144738/https://medicaid.georgia.gov/document/document/modified-1332-waiver/download |
| Maine | 2022 | Initial | https://web.archive.org/web/20250306144830/https://www.maine.gov/pfr/insurance/sites/maine.gov.pfr.insurance/files/inline-files/maine-section-1332%20waiver-complete-application-02-10-2022.pdf |
| Maryland | 2018 | Initial | https://web.archive.org/web/20250306144902/https://www.marylandhbe.com/wp-content/uploads/2018/08/Maryland_1332_State_Innovation_Waiver_to_Establish_a_State_Reinsurance_Program_UPDATED_August_15_2018.pdf |
| Minnesota | 2017 | Initial | https://web.archive.org/web/20250306145405/https://www.cms.gov/cciio/programs-and-initiatives/state-innovation-waivers/downloads/minnesota-section-1332-waiver.pdf |
| New Hampshire | 2020 | Initial | https://web.archive.org/web/20230215100827/https://www.nh.gov/insurance/lah/documents/nh-section-1332-waiver-draft.pdf |
| New Jersey | 2018 | Initial | https://web.archive.org/web/20250306145323/https://www.nj.gov/dobi/division_insurance/section1332/180702finalwaiverapplication.pdf |
| New Jersey | 2023 | Renewal | https://web.archive.org/web/20250306145235/https://www.nj.gov/dobi/division_insurance/section1332/2023extension/revisedextensionapplication.pdf |
| North Dakota | 2019 | Initial | https://web.archive.org/web/20250201173135/https://www.insurance.nd.gov/sites/www/files/documents/Health%20Care%20Reform/Final%20North%20Dakota%201332%20Waiver%20Application.pdf |
| Oregon | 2017 | Initial | https://web.archive.org/web/20250306144842/https://healthcare.oregon.gov/DocResources/1332-application.pdf |
| Oregon | 2022 | Renewal | https://web.archive.org/web/20250306145333/https://dfr.oregon.gov/business/reg/health/Documents/reinsurance-program/innovation-waiver-2023-2027-app.pdf |
| Pennsylvania | 2020 | Initial | https://web.archive.org/web/20250306151810/https://www.pa.gov/content/dam/copapwp-pagov/en/insurance/documents/coverage/documents/pennsylvania%201332%20reinsurance%20waiver%20final%20application.pdf |
| Rhode Island | 2019 | Initial | https://web.archive.org/web/20250306144818/https://healthsourceri.com/wp-content/uploads/190708_FinalApplicationPackage.pdf |
| Rhode Island | 2024 | Renewal | https://web.archive.org/web/20250306145311/https://healthsourceri.com/wp-content/uploads/20240709_FinalRenewalApplication_Aug-Update.pdf |
| Wisconsin | 2018 | Initial | https://web.archive.org/web/20250306144924/https://oci.wi.gov/Documents/Regulation/ATTACHMENT%201%20Wisconsin_1332%20Actuarial%20Economic%20Analysis%20Report_04_17_2018.pdf |
| Wisconsin | 2022 | Renewal | https://web.archive.org/web/20250306144942/https://oci.wi.gov/Documents/AboutOCI/1332_WaiverExtensionApplicationWI.pdf |
